# Toxicarioside A Inhibits Tumor Growth and Angiogenesis: Involvement of TGF-β/Endoglin Signaling

**DOI:** 10.1371/journal.pone.0050351

**Published:** 2012-11-28

**Authors:** Feng-ying Huang, Wen-li Mei, Yue-nan Li, Guang-hong Tan, Hao-fu Dai, Jun-li Guo, Hua Wang, Yong-hao Huang, Huan-ge Zhao, Song-lin Zhou, Ying-ying Lin

**Affiliations:** 1 Hainan Provincial Key Laboratory of Tropical Medicine, Hainan Medical College, Haikou, People's Republic of China; 2 Institute of Tropical Bioscience and Biotechnology, Chinese Academy of Tropical Agricultural Sciences, Haikou, People's Republic of China; Mayo Clinic College of Medicine, United States of America

## Abstract

Toxicarioside A is a cardenolide isolated mainly from plants and animals. Emerging evidence demonstrate that cardenolides not only have cardiac effects but also anticancer effects. In this study, we used *in vivo* models to investigate the antitumor activities of toxicarioside A and the potential mechanisms behind them. Murine colorectal carcinoma (CT26) and Lewis lung carcinoma (LL/2) models were established in syngeneic BALB/c and C57BL/6 mice, respectively. We found that the optimum effective dose of toxicarioside A treatment significantly suppressed tumor growth and angiogenesis in CT and LL/2 tumor models *in vivo*. Northern and Western blot analysis showed significant inhibition of endoglin expression in toxicarioside A-treated human umbilical vein endothelial cells (HUVECs) *in vitro* and tumor tissues *in vivo*. Toxicarioside A treatment significantly inhibited cell proliferation, migration and invasion, but did not cause significant cell apoptosis and affected other membrane protein (such as CD31 and MHC I) expression. In addition, TGF-β expression was also significantly inhibited in CT26 and LL/2 tumor cells treated with toxicarioside A. Western blot analysis indicated that Smad1 and phosphorylated Smad1 but not Smad2/3 and phosphorylated Smad2/3 were attenuated in HUVECs treated with toxicarioside A. Smad1 and Smad2/3 signaling remained unchanged in CT26 and LL/2 tumor cells treated with toxicarioside A. Endoglin knockout by small interfering RNA against endoglin induced alternations in Smad1 and Smad2/3 signaling in HUVECs. Our results indicate that toxicarioside A suppresses tumor growth through inhibition of endoglin-related tumor angiogenesis, which involves in the endoglin/TGF-β signal pathway.

## Introduction

*Antiaris toxicaria* is well known as a poison for arrows, darts, and blowdarts in many countries [1], [2]. In China, this plant is known as “arrow poison wood” because its latex contains a complex, toxic mixture of cardenolide glycosides. *Antiaris toxicaria* grows widely throughout many tropical areas in Southeast Asia. In China, it is mainly distributed in the warmer southern and eastern areas, such as Guangxi, Guangdong, Yunnan, and Hainan provinces. Early studies of the toxic agents of this plant in Indonesia and Malaysia have resulted in the isolation of several kinds of cardenolides from the latex, seeds, and stem [3], [4]. Traditionally, cardenolides have generally been accepted in the treatment of congestive heart failure and as anti-arrhythmic agents [5]–[7]. However, recent studies have demonstrated that certain cardenolides extracted from some plants and animals are involved in complex cell signal transduction mechanisms that may have important consequences in blocking tumor cell proliferation and inducing tumor apoptosis [8]–[15]. In recent years, our research group has isolated three new cytotoxic cardenolides from the latex of *Antiaris toxicaria*. These have been shown to possess significant cytotoxicity against K562, SGC-7901, SMMC-7721, and HeLa cell lines [16], [17]. In addition, we also found that toxicarioside A has the capabilities of inhibiting NF-κB/bFGF and endoglin/TGF-β signaling pathways in a gastric cancer and bone marrow stromal cell lines, respectively [18], [19].

Previous studies have demonstrated that endoglin is a homodimeric transmembrane glycoprotein that can bind specifically to transforming growth factor β1 (TGF-β1), TGF-β3, activin A, and several bone morphogenic proteins after incorporation with one of two transmembrane serine-threonine kinases, TGF-β receptor I or TGF-β receptor II [20]. Endoglin is constitutively phosphorylated, but it is not an active signaling molecule and only works as an auxiliary component by formation of a heteromeric complex with the TGF-β receptor, by which it modulates the signaling of distinct TGF-β receptor I isotypes known as activin-receptor-like kinase (ALK)-1 and ALK-5 [21], [22]. After formation of the activated heteromeric complex, endoglin modulates an intracellular signaling cascade by which specific Smad proteins are activated and further signals are transduced into the nucleus. There they regulate the transcription of a series of genes involved in maintaining normal physiologic functions, such as cell proliferation, apoptosis, cell motility, cell adhesion, and tumor angiogenesis [23].

In addition, accumulating evidence has demonstrated that endoglin is over-expressed and up-regulated in tumor-associated angiogenic vasculature relative to normal tissue vasculature [24], [25]. Immunotherapies with anti-endoglin monoclonal antibody, DNA and protein vaccines against endoglin have been shown to inhibit tumor growth and metastasis by suppressing endoglin-related angiogenesis *in vivo* [26]–[28]. Because we have isolated three new cytotoxic cardenolides as described above, we therefore think it is reasonable to conceive that these cytotoxic cardenolides may potentially have some ability to inhibit tumor growth by suppressing angiogenesis. In the present study, we examined whether toxicarioside A, a cardenolide isolated from *Antiaris toxicaria*, could inhibit tumor growth and its relationship with the endoglin. Our results indicate that toxicarioside A can suppress tumor growth by inhibiting angiogenesis, which involves in the endoglin/TGF-β signal pathway.

## Materials and Methods

### Isolation and identification of toxicarioside A

**Figure 1:**
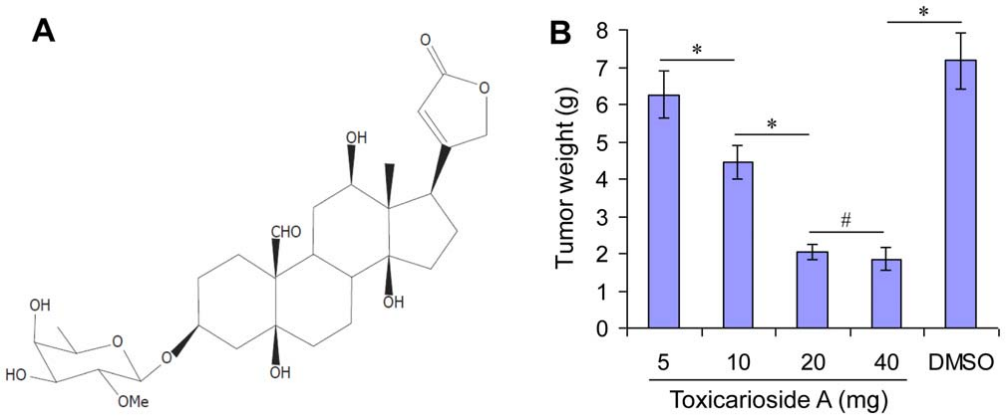
Structure and optimum effective dose of toxicarioside A. (**A**) Structure of toxicarioside A isolated from *Antiaris toxicaria* in Hainan, China. (**B**) CT26-bearing BABL/c mice were randomly divided into five groups (n = 5) and i.v. injected with the indicated doses of toxicarioside A (5, 10, 20, and 40 mg/kg in 100 µL DMSO) and 100 µL DMSO every 3 days for 18 days. The results show tumor weight on day 18, indicating that 20 mg/kg is the optimum dose. Data are expressed as mean ± SEM, *P<0.01 or less, ^#^ *P*>0.05.

Latex from *Antiaris toxicaria* was collected from Lingshui County in Hainan Province, China, in November 2005. The plant was validated by Professor Zhu-nian Wang in the Institute of Crops Genetic Resources, Chinese Academy of Tropical Agricultural Sciences. The voucher specimen was numbered AN200511 and deposited in the Institute of Tropical Bioscience and Biotechnology, Chinese Academy of Tropical Agricultural Sciences. Toxicarioside A was isolated from the fractionation of the 60% ethanol extract of the latex of *Antiaris toxicaria*, as previously reported [15]. Its structure was elucidated by comprehensive analysis of 1D and 2D NMR spectra (Figure 1A). The resultant toxicarioside A was dissolved in DMSO in a stock concentration (1 mg/ml) for subsequent experiments. The field studies in this study were permitted by Institute of Tropical Bioscience and Biotechnology, Chinese Academy of Tropical Agricultural Sciences. The plant samples were delicately collected to avoid causing death.

### Cell culture

Murine colorectal carcinoma cell line CT26 (CT26) cells, Lewis lung carcinoma (LL/2) cells were purchased from the American Type Culture Collection (ATCC). The human umbilical vein endothelial cells (HUVECs) were isolated and cultured as we reported previously [29]. The tumor cells and HUVECs were cultured in DMEM, RPMI1640, or F-12K media (Gibical) and supplemented with 10% fetal bovine serum, 2 mmol/L glutamine, 100 IU/mL penicillin, and 100 μg/ml streptomycin at 37°C in a humidified atmosphere with 5% CO_2_. For analysis of endoglin expression and Smad signaling in HUVEC, 100 ng/mL recombinant TGF-β1 (eBioscience) was added to the F-12K medium. Cells from the logarithmic phase were used for subsequent experiments.

**Figure 2:**
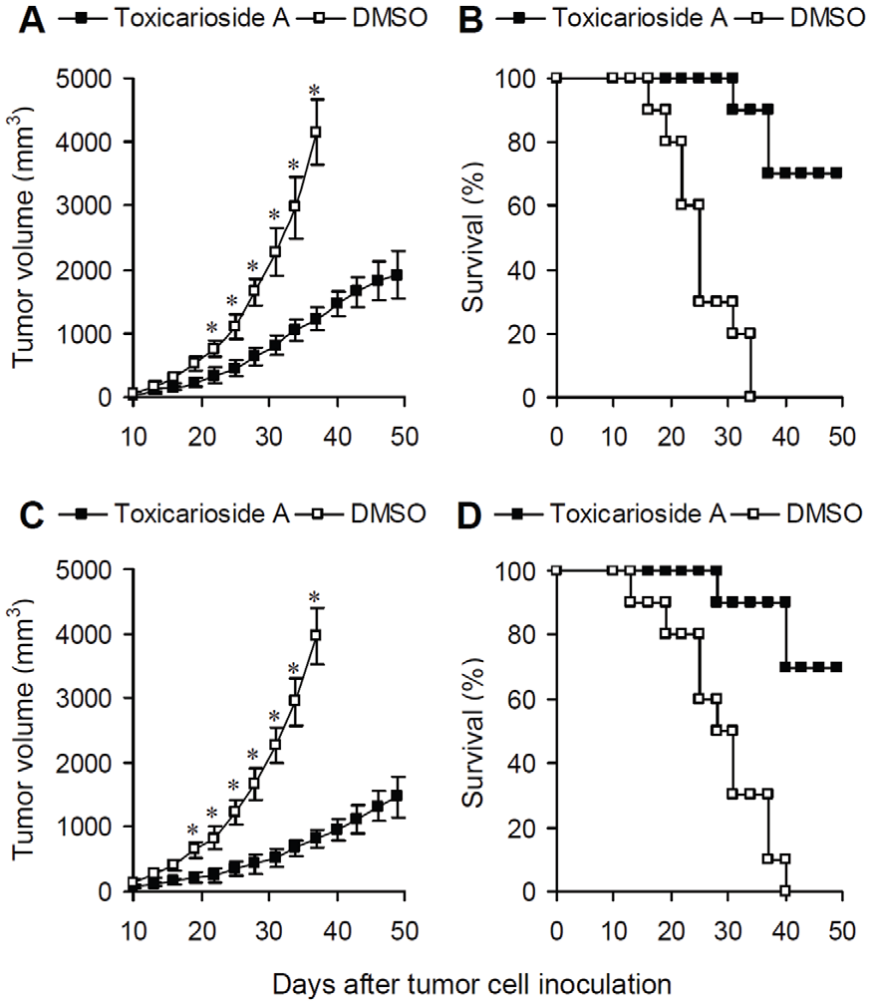
Inhibition of tumor growth by toxicarioside A *in vivo*. CT26 (A and B) and LL/2(C and D) model mice were randomly divided into two groups (*n* = 10) and i.v. injected with toxicarioside A (20 mg/kg in 100 µL DMSO) and DMSO 100 µL every 3 days for 21 days. (**A** and **C**) Tumor volumes at different points in time in mice treated with toxicarioside A or DMSO. (**B** and **D**) Survival rates at different points in time for mice treated with toxicarioside A or DMSO. Data are expressed as means ± SEM, * *P*<0.05 or less.

### Establishment of tumor models

Female mice at 6 to 8 weeks of age were used for establishment of tumor models. Colorectal carcinoma CT26 model was established in BALB/c mice, and a Lewis lung carcinoma model was established in C57BL/6 mice. To established tumor models, mice were injected s.c. with 2×10^6^ corresponding tumor cells in the right flank. When the tumor masses were palpable, the mice were randomly divided into groups, treated with experimental or control agents, and observed. The animal protocols in this study were approved by the College's Animal Care and Use Committee (approval ID: HNMCE10012-7). Tumor size was monitored and evaluated by measuring the longest dimension (length) and shortest dimension (width) at 3-day intervals with a dial caliper, and tumor volume was calculated according to the following formula: Tumor volume  = 0.52× length × (width)^2^. Survival curves were constructed according to the Kaplan-Meier method.

**Figure 3:**
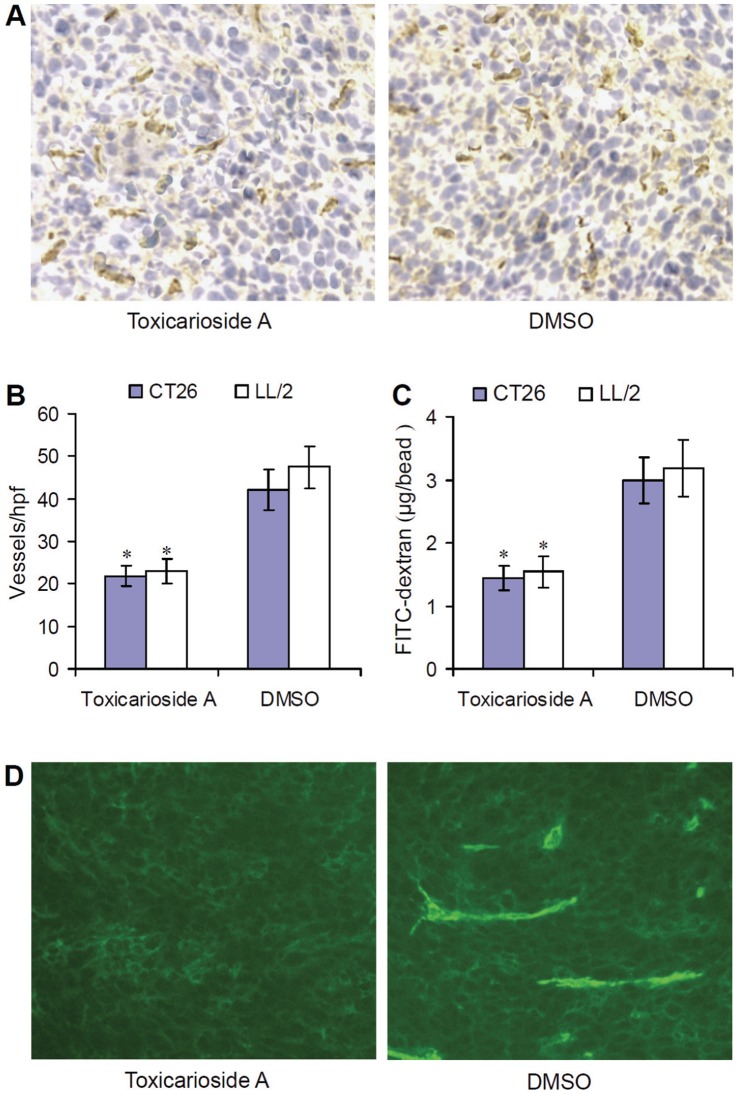
Inhibition of tumor angiogenesis and endoglin expression by toxicarioside A *in vivo*. (**A**) Frozen sections of tumor tissues were tested by immunohistochemical analysis with a monoclonal antibody against CD31 and the representative high-power field (hpf) in the sections under a microscope at 200× magnification. (**B**) The tumor vasculatures were quantified by counting the microvessels per hpf. (**C**) Quantitative analysis of the alginate implants. Alginate beads containing 1×10^5^ tumor cells were implanted s.c. into the backs of mice. Mice were then treated with toxicarioside A or DMSO. Beads were surgically removed 14 days later and the FITC–dextran absorbed in the beads was quantified by a fluorescent ELISA reader. Data are expressed as means ± SEM, * *P*<0.001 or less, relative to the DMSO-treated group. (**D**) Shown are the representative images of endoglin expression *in situ* on the tumor vessels. Frozen sections of tumor tissues treated with toxicarioside A or DMSO were incubated with an antibody against endoglin and a second FITC-conjugated antibody was used to stain the sections. Slides were examined by a fluorescence microscopy at 200× magnification.

### Determination of optimum effective dose

CT26-bearing BABL/c mice were randomly divided into five groups (n = 5 for each group). Treatment was begun when tumor volume was about 90 mm^3^. These mice were injected i.v. with various does of toxicarioside A (5, 10, 20, or 40 mg/kg in 100 µL DMSO) and DMSO (100 µL). All these reagents were given every 3 days for 18 days. The mice were killed on day 21, and tumor tissues were excised and weighed. The optimum effective dose of toxicarioside A for subsequent experiments was determined according to tumor weight.

**Table 1:** The effects of toxicarioside A (Tox A) on cell proliferation.

Groups/Cell	Dose (μg/mL)	24 h		48 h	
		OD value	Inhibition (%)	OD value	Inhibition (%)
HUVECs					
DMSO	0.0	0.928±0.042	0.00±0.00	0.905±0.033	0.00±0.00
Tox A	0.5	0.795±0.087	14.81±5.49	0.724±0.053	20.39±5.72
	1.5	0.633±0.063	31.64±5.91*	0.561±0.083	38.97±4.76*
	4.5	0.580±0.065	37.27±4.58*	0.455±0.062	49.86±4.37**
	9.0	0.337±0.088	62.75±7.38**	0.316±0.052	65.51±6.48**
CT26					
DMSO	0.0	0.894±0.033	0.00±0.00	0.887±0.049	0.00 ± 0.00
Tox A	0.5	0.771±0.046	13.27±5.33	0.719±0.062	19.06 ± 6.44
	1.5	0.607±0.052	32.46±6.09*	0.559±0.071	37.03±6.08*
	4.5	0.546±0.059	39.03±5.82*	0.427±0.055	51.41±5.07**
	9.0	0.421±0.076	53.28±6.44**	0.322±0.061	63.94±5.03**
LL/2					
DMSO	0.0	0.903±0.029	0.00±0.00	0.839±0.037	0.00±0.00
Tox A	0.5	0.795±0.037	12.17±4.29	0.697±0.053	17.94±4.25
	1.5	0.624±0.041	29.95±5.12*	0.522±0.059	36.95±5.73*
	4.5	0.561±0.053	37.99±6.34*	0.403±0.062	52.07±6.11**
	9.0	0.455±0.068	49.82±7.01**	0.307±0.079	63.92±6.54**

Data of six independent experiments were expressed as mean ± SEM. * *P*<0.05, ** *P*<0.01 versus DMSO control group.

### Observations of antitumor activities and possible side effects

Tumor-bearing mice inoculated with CT26 and LL/2 cells were divided into two groups (n = 10 for each group). One group of mice was i.v. injected with optimum does of toxicarioside A (dissolved in 100 µL DMSO) once every 3 days. Another group of mice was i.v. injected with DMSO 100 µL once every 3 days. The tumor volumes and survival time were observed and recorded. The mice were killed when they became moribund and the date of death was recorded to calculate the survival time. In addition, tumor tissues were also excised, fixed in 10% formalin and frozen at −80°C for detections of microvessel density.

Moreover, potential toxicities in the mice treated with toxicarioside A were also investigated. Gross measures including weight loss, life span, ruffling of fur, feeding and behavior were observed. Tissues of major organs such as liver, kidney, heart, lung, spleen and brain were collected in the end of the experiment and fixed in 10% neutral buffered formalin solution and embedded in paraffin. Sections of 3 to 5 μm were stained with HE and tissue structures and cellular morphology were observed under microscope.

### Detection of microvessel density and endoglin expression in vessels

For microvessel density (MVD) analysis, frozen sections of tumor tissues were fixed in acetone, incubated, and stained with an antibody reactive to CD31, as performed previously [26]. The sections were then stained with labeled streptavidin biotin reagents (Dako LSAB kit, peroxidase; Dako). Vessel density was determined by counting the number of microvessels per high-power field (hpf) in the sections under a microscope (80i, Nikon) at 200× magnification. Images were captured with a digital photography system (DP72, Olympus).

Endoglin expression *in situ* on the tumor vessels was detected by immunohistochemistry. Frozen sections of tumor tissues were fixed in acetone, incubated with a monoclonal rabbit IgG antibody against endoglin (Santa Cruz) and washed with PBST (0.05% Tween 20 in PBS). Thereafter, a second goat FITC-conjugated antibody against rabbit IgG (Sigma) was used to stain the sections and washed with PBST. Slides were examined by fluorescence microscopy (80 i, Nikon) and images were captured as above at 200× magnification.

### Proliferation, invasion and migration assays

MTT assay was used to determine cell proliferation. HUVECs and both CT26 and LL/2 tumor cells in logarithmic growth were trypsinized and harvested, and then the cells were seeded onto a 96-well plate. After 24 h, fresh RPMI 1640 or DMEM medium containing different concentrations of toxicarioside A was added at 100 μL per well respectively and each concentration has 6 replicate wells. After incubation for different time intervals, 10 μL of MTT (5 mg/mL) was added to each well and the cells were further incubated at 37°C for 4 hours. Then the supernatant was removed and 100 μL DMSO was added into each well. The absorbance (OD value) at wavelength of 490 nm was measured with a microplate reader (Bio-Tek EXL808).

Invasion assay was performed in a 24-well transwell chamber (Corning, Lowell) as previously described [19]. In brief, each transwell chamber was coated with 15 μg Matrigel, 5×10^4^ cells were seeded to per-coated filters in 200 μl of serum-free medium containing different concentrations of toxicarioside A in triplicate, and the lower parts of the chambers were filled with 500 μL of medium containing 10% FBS. The plates were incubated in a 5% CO_2_ humidified incubator at 37°C for 24 h. After the cells on the upper surface were gently removed with a cotton swab, the filters were fixed with 95% alcohol for 15–20 min and stained with HE for 15 min, and then the cells on the lower surface of the filters were quantified under a microscope at 200× magnification. Migration assay was performed by using method similar to the transwell invasion chamber to assess the cell motility, except that transwell chamber was not coated with Matrigel.

### Detection of cell apoptosis *in vitro*

A TUNEL-based apoptosis detection was performed using a TiterTACS In Situ Detection Kit (Trevigen) as described in a previous study [30]. In brief, cells were seeded in 96-well plates and incubated for 16 h in the presence or absence of tested drugs at a various concentration. Thereafter, cells were fixed and nick-end labeled as recommended by the manufacturer's protocol. The absorbance at 450 nm (A450 nm), corresponding to the number of nick-ends, was normalized to the number of cells, as evaluated from crystal violet staining (A540 nm). To ensure clear representation, all A450 nm/A540 nm signals were normalized to the signal obtained using an unlabeled sample (cells treated with DMSO without nick-end labeling) as negative control. For a positive control, nuclease-treated cells were treated with TACS nuclease after fixation and then nick-end labeled.

### Alginate encapsulation assay

Alginate-encapsulated tumor cell assays were performed as previously described [31]. Briefly, CT26 cells were resuspended in a 1.5% solution of sodium alginate and added dropwise into a swirling 37°C solution of 250 mM calcium chloride. Alginate beads were formed containing approximately 1×10^5^ tumor cells per bead. Experimental mice were then anesthetized, and four beads were implanted subcutaneously into an incision made on the dorsal side. Incisions were closed with surgical clamps. After 14 days, mice were injected intravenously with 100 µL of a 100 mg/kg FITC-dextran solution (Sigma). Beads were surgically removed and FITC-dextran was quantified against a standard curve of FITC-dextran using a fluorescent ELISA reader (ELX808IU, Bio-Tek).

### RNA isolation and Northern blot analysis

Total RNA was isolated directly from cultured cells and tumor tissues using TRIzol reagent (Gibco-BRL/Invitrogen) as recommended by the manufacturer's instructions. For Northern blot analysis, RNA was transferred to Hybond N^+^ membranes and then hybridized with full-length cDNA probes for murine endoglin and β-actin in PerfectHyb Plus hybridization buffer (Sigma-Aldrich) according to the manufacturer's instructions. Digital images were acquired and analyzed with a gel imaging system (Bio-Rad Gel Doc1000, Bio-Rad). The resultant mRNA levels were compared to β-actin and expressed as percentages of β-actin.

### Western blot analysis

Western blot analysis was performed as described previously [31]. In brief, lysates of cells treated with toxicarioside A or DMSO and tumor-tissue homogenate proteins from mice treated with toxicarioside A or DMSO were separated using 12% SDS-PAGE. Gels were further transported onto a polyvinylidene difluoride membrane (Bio-Rad) by a mini trans-blot system (Bio-Rad). The membrane blots were blocked at 4°C in 5% nonfat dry milk, washed, and probed with antibodies against corresponding target molecules at 1∶500 (endoglin, TGF-β, Smad1, Smad2/3, pSmad1, and pSmad2/3, all purchased from Santa Cruz, Abcam, Innovations or Cell Signaling Technology). They were then detected using an enhanced chemiluminescence system (Amersham) as previously reported [32]. Digital images were acquired and analyzed with a gel imaging system (Bio-Rad Gel Doc1000, Bio-Rad). The resultant protein level was compared to β-actin and is here expressed as percentage of β-actin.

### Flow cytometry

To detect the membrane protein CD31 and MCH I on HUVEC and on CT26 and LL/2 tumor cells, cells were blocked with monoclonal antibodies against CD31 or MHC I (BD Biosciences). Thereafter, cells were stained with a FITC-conjugated second antibody and analyzed on a FACSCalibur (BD Biosciences) using CellQuestPro software (BD Biosciences).

### Preparation of small interfering RNA against endoglin and cell infection

Small interfering RNA (siRNA) against endoglin were prepared as previously reported [33], [34]. Briefly, siRNA duplexes against murine endoglin were designed and synthesized by a commercial biotechnological company (Takara, China). Scrambled siRNA was also obtained from Takara and was used as the control siRNA. HUVECs were transfected with the siRNA duplexes by electroporation using an electroporation system (Bio-Rad Gene Pulser II). For transfection, 2×10^6^ cells were resuspended in 1 mL electroporation buffer with 200 nmol/L dsRNA. Thereafter, the resuspended cells were transferred to a Gene Pulser cuvette (Bio-Rad) and electroporated (1 pulse, 0.2 kV, 0.3 μF, 73.8 ms). The electroporated cells were then combined, mixed with 4 mL cultured medium, and plated onto four wells of a six-well culture plate at a concentration of 1×10^6^ per well. All cultures were incubated at 37°C in a humidified air/CO_2_ (95∶5, v/v) atmosphere for the duration of the experiment. At 24 h posttransfection, the toxicarioside A and DMSO were added into the cultured medium, and the mixture was allowed to incubate for an additional 48 h. The cultured cells were collected and used for Western blot analysis as above.

### Statistical analysis

An unpaired Student's *t*-test was used. Survival curves were constructed according to the Kaplan-Meier method and statistical significance was determined by the log-rank test. *P* values <0.05 were considered significant. Error bars represent SEM unless otherwise indicated.

## Results

### Optimum effective dose

CT26-bearing BABL/c mice were treated with toxicarioside A at different doses once every 3 days. Eighteen days after inoculation, the mice were killed and tumor masses were removed and weighed. Our results showed that toxicarioside A affected tumor size in a dose-dependent manner (Figure 1B). The mice treated with 5 and 10 mg/kg toxicarioside A showed some inhibition of tumor growth, but the mice treated with 20 and 40 mg/kg showed similar significant levels of inhibition of tumor growth (Figure 1B). Therefore, 20 mg/kg toxicarioside A was considered the optimum effective dose and used for subsequent *in vivo* experiments to investigate the anti-tumor and anti-angiogenesis activities.

### Antitumor effects and side effects

The antitumor activities induced by toxicarioside A were observed in tumor models *in vivo*. CT26-bearing BABL/c mice (Figs. 2A and 2B) and LL/2-bearing C57BL/6N mice (Figs. 2C and 2D) were treated with toxicarioside A and DMSO respectively. Tumor volumes (Figure 2A and 2C) and survival rates (Figs. 2B and 2D) were observed. Relative to mice treated with DMSO, the tumor volumes in the mice treated with toxicarioside A were significantly decreased and the survival rates were high, suggesting significant inhibition of tumor growth and prolonged survival time in the mice treated with toxicarioside A (Figure 2, *P*<0.05 or less after day 23).

In the present study, no significant adverse consequences were found in gross measures such as ruff ling of fur, weight loss, behavior and life span during the experiment. At the end of the experiment, the major organs in each group were collected and HE stained for microscopic examination. There were not significant pathologic changes in any major organ such as liver, kidney, lung, spleen, brain or heart in the mice treated with toxicarioside A when compared to the control mice (data not shown).

### Inhibition of angiogenesis *in vivo*

Angiogenesis within tumor masses was evaluated by counting the number of microvessels on the sections stained with an antibody against to CD31. The average number of vessels per high-power field (hpf) in mice treated with toxicarioside A was significantly decreased relative to those of mice treated with DMSO (Figure 3A and 3B, *P*<0.001), 21.86±2.38 versus 42.03±4.83 in the CT26 model and 22.97±2.96 versus 47.41±5.05 in the LL/2 model, respectively. In addition, the inhibition of angiogenesis in the mice treated with toxicarioside A was also confirmed in alginate encapsulation assay. CT26 and LL/2 tumor cells were encapsulated in alginate beads and implanted subcutaneously in corresponding mice. Angiogenesis was then quantified by measuring the uptake of FITC-dextran into the beads. Similar results were found in the alginate encapsulation assay FITC-dextran uptake was found to be significantly reduced in mice treated with toxicarioside A relative to mice treated with DMSO (Figure 3C, *P*<0.001), 1.44±0.19 versus 2.99±0.36 (μg) in the CT26 model and 1.54±0.24 versus 3.19±0.45 (μg) in the LL/2 model.

### Effects on HUVEC, CT26 and LL/2 tumor cell proliferation, migration, invasion and apoptosis

HUVEC, CT26 and LL/2 cells were treated with toxicarioside A at different concentrations (0.5, 1.5, 4.5, 9.0 μg/ml) for 24–48 h, the results of MTT assay demonstrated that toxicarioside A treatment caused significantly inhibition of cell proliferation on HUVECs, CT26 and LL/2 tumor cells *in vitro*. The inhibition effects on HUVECs, CT26 and LL/2 cells caused by toxicarioside A were found to be both dose and time dependent; the OD value decreased gradually, and the inhibitory rate increased gradually in HUVECs and both CT26 and LL/2 tumor cells (Table 1).

Similar results as MTT assay were found in the migration and invasion assays. From the results of Transwells *in vitro* (Table 2), addition of toxicarioside A to the conditioned medium in the upper chamber suppressed the migration and invasion of HUVECs and both CT26 and LL/2 tumor cells also in a dose-dependent manner. When compared with the DMSO control group, treatment with toxicarioside A at doses of 1.5, 4.5, and 9.0 μg/ml caused significantly inhibition of cell migration and invasion on HUVECs and both CT26 and LL/2 tumor cells (Table 2).

**Table 2:** The effects of toxicarioside A (Tox A) on cell migration and invasion.

Cells/Groups	Dose (μg/mL)	Migration		Invasion	
		Cell number	Inhibition (%)	Cell number	Inhibition (%)
HUVECs					
DMSO	0.0	82.73±9.94	0.00±0.00	71.98±9.37	0.00±0.00
Tox A	0.5	70.38±8.86	15.06±7.29	61.47±7.52	14.70±5.33
	1.5	58.39±7.75	29.61±8.90*	52.84±6.02	26.85±7.41*
	4.5	46.55±6.09	43.07±9.77*	42.39±5.53	41.36±8.37*
	9.0	37.21±5.23	55.63±11.42**	32.51±5.07	55.02±9.62**
CT26					
DMSO	0.0	94.27±11.03	0.00±0.00	69.43±8.58	0.00±0.00
Tox A	0.5	82.91±10.27	12.18±5.33	58.47±6.92	15.37±6.71
	1.5	69.46±9.28	26.83±7.42*	50.22±5.37	27.85±8.07*
	4.5	56.53±8.99	40.61±8.06*	41.56±6.02	40.58±9.01*
	9.0	42.89±7.55	54.88±9.67**	29.81±6.03	57.34±9.35**
LL/2					
DMSO	0.0	78.24±8.38	0.00±0.00	86.33±9.08	0.00±0.00
Tox A	0.5	69.45±7.21	11.53±5.07	71.01±8.23	17.24±4.86
	1.5	52.06±6.04	33.01±7.32*	60.68±7.25	29.25±6.03*
	4.5	41.09±5.27	47.25±8.03**	51.05±7.01	40.97±8.01*
	9.0	34.88±4.91	54.97±9.85**	40.44±6.57	53.29±8.94**

Data of six independent experiments were expressed as mean ± SEM. * *P*<0.05, ** *P*<0.01 versus DMSO control group.

**Figure 4:**
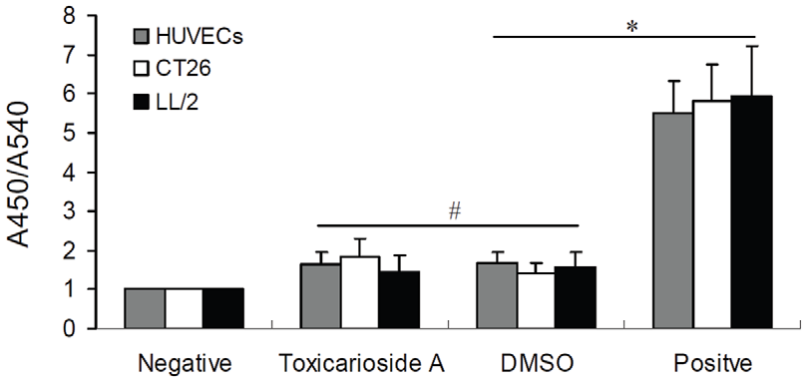
No affect on cell apoptosis *in vitro*. HUVECs, CT26, and LL/2 cells were treated with toxicarioside A or DMSO for 24 h. Thereafter, cells were fixed and nick-end labeled. The number of nick-ends (A450 nm) was divided by the number of cells as evaluated by crystal violet staining (A540 nm). A450/A540 nm signals were normalized to the signal obtained in unlabeled cells. These cells became the negative group. Cells in the positive group were treated with TACS nuclease after fixation and then nick-end labeled. Toxicarioside A treatment did not cause significant cell apoptosis. The graph shows the results of a representative experiment run in triplicate. Data are expressed as mean ± SEM. * *P*<0.001 and ^#^ *P*>0.05.

A TUNEL-based assay was performed to determine the number of nick-ends generated as a result of DNA fragmentation during apoptosis. The results performed in HUVEC, CT26, and LL/2 cells are shown in Figure 4. Compared with the DMSO control group, the numbers of nick-ends in toxicarioside-A-treated HUVEC, CT26, and LL/2 cells were similar to those observed in corresponding unlabeled negative cells, indicating that toxicarioside A dose not have significantly anti-apoptotic effects on HUVEC, CT26, and LL/2 cells (Figure 4).

### Inhibition of endoglin expression *in vitro* and *in vivo*

**Figure 5:**
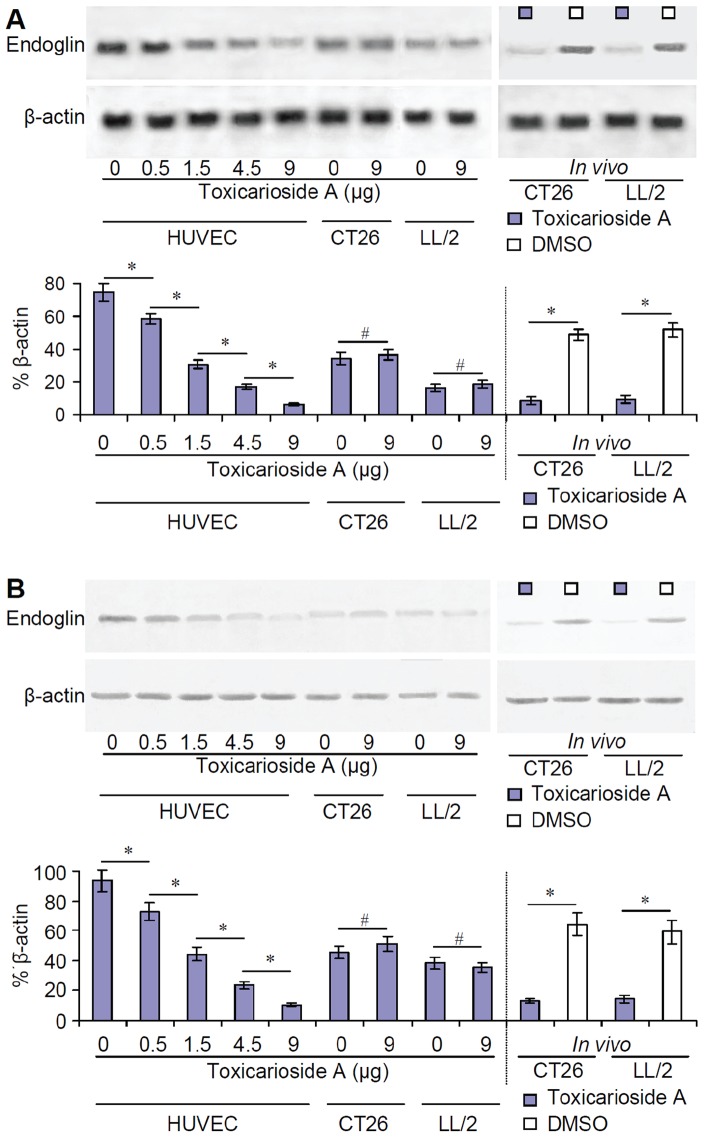
Inhibition of endoglin expression by toxicarioside A *in vitro* and *in vivo*. Endoglin mRNA and protein in cultured cells and tumor tissues were detected by Northern and Western blot. (**A**) The results of Northern blot analysis. Expression of endoglin mRNA was inhibited by toxicarioside A in dose-dependent manner in HUVECs and in tumor tissues treated with toxicarioside A but not in CT26 or LL/2 tumor cells. (**B**) The results of Western blot analysis. Expression of endoglin protein was similar to that observed in Northern blot analysis. Data are expressed as mean ± SEM, *P<0.01 or less, ^#^ *P*>0.05.

Endoglin mRNA was detected by Northern blot analysis. HUVECs, CT26 cells, and LL/2 cells were cultured in different concentrations of toxicarioside A. Total mRNA was isolated for Northern blot analysis. The results of Northern blot analysis showed that toxicarioside A inhibited endoglin expression in a dose-dependent manner in the HUVECs but not in the CT26 or LL/2 tumor cells. Doses of 0.5 to 9 μg induced significant inhibition of endoglin expression relative to untreated cells and other doses (Figure 5A, left, *P*<0.01). Tumor tissues were also subjected to total mRNA isolation and Northern blot analysis. Figure 5A (right) shows that endoglin mRNA expression in both CT26 and LL/2 tumor tissues treated with toxicarioside A were significantly suppressed relative to the tumor tissues treated with DMSO (*P*<0.01).

Endoglin protein was isolated by SDS-PAGE and detected by Western blot analysis. Results were similar to those of Northern blot analysis (Figure 5B). Endoglin protein expression was also observed in a dose-dependent pattern only in the HUVECs, not in CT26 or LL/2 tumor cells (Figure 5B, left). *In vivo* experiments indicated endoglin protein in the CT26 and LL/2 tumor tissues treated with toxicarioside A but not in the tumor tissues treated with DMSO (Figure 4B, right). Statistical analysis indicated that significant differences existed between HUVECs treated with various doses of toxicarioside A and between the tumor tissues treated with toxicarioside A and DMSO (Figure 5B, *P*<0.01). These *in vitro* and *in vivo* results suggest that toxicarioside A treatment can significantly inhibit endoglin expression in tumor angiogenesis-related cells, such as HUVECs, but not in tumor cells (CT26 and LL/2).

In addition, endoglin expression was also detected *in situ* in the tumor vessels by immunofluorescence. Sections of tumor tissue were stained using monoclonal endoglin antibody and a FITC-conjugated second antibody. The slides were then examined by fluorescence microscopy at 200× magnification. There were no obvious fluorescent signals in the sections from the tumor tissues treated with toxicarioside A, but strong fluorescent signals were found in the sections of tumor tissues treated with DMSO (Figure 3D), suggesting that *in situ* endoglin expression on the tumor vessels was also suppressed by toxicarioside A treatment.

### Effects on expression of other cell membrane proteins

**Figure 6:**
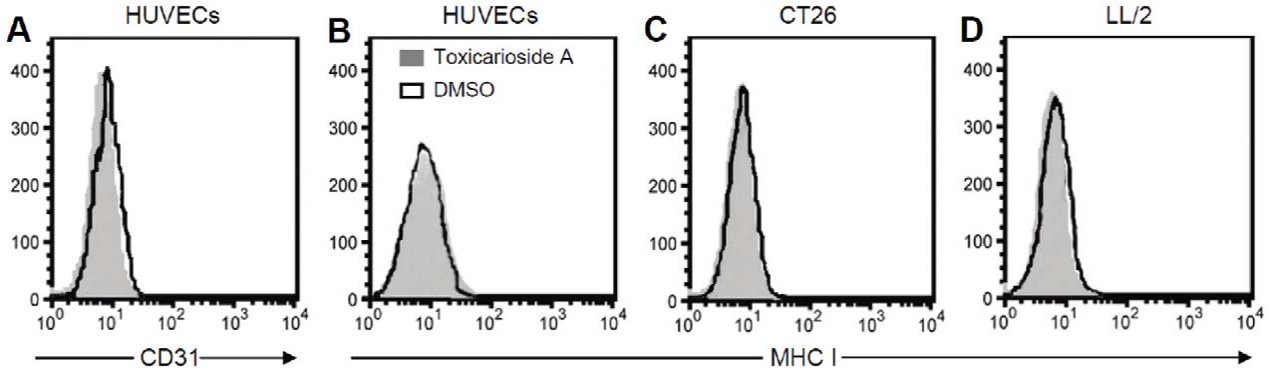
No affect on membrane protein expression. HUVECs, CT26 and LL/2 cells were treated with toxicarioside A (4.5 μg/mL) or DMSO for 24 h. Thereafter, cells were stained with anti-CD31 or anti-MHC I and analyzed by flow cytometry. Shown are representative histograms of cells treated with toxicarioside A (gray) and DMSO (solid black line).

As we know, endoglin is a membrane protein. To determine whether toxicarioside A affects the membrane protein expression, CD31 and MHC I antigen were chosen as representative membrane proteins on HUVECs and tumor cells. Flow cytometric analysis revealed that CD31 only expressed on HUVECs, but MHC I expressed on HUVECs and both CT26 and LL/2 tumor cells (Figure 6). Moreover, the expression levels of CD31 on HUVECs and expression levels of MHC I on HUVECs and on both CT26 and LL/2 tumor cells were similar between cells treated with toxicarioside A and DMSO (Figure 6). These results indicate that toxicarioside A can suppress endoglin expression, but does not affect other membrane protein (such as CD31 and MHC I) expression in HUVECs, CT26 and LL/2 cells.

### Inhibition of TGF-β signaling

**Figure 7:**
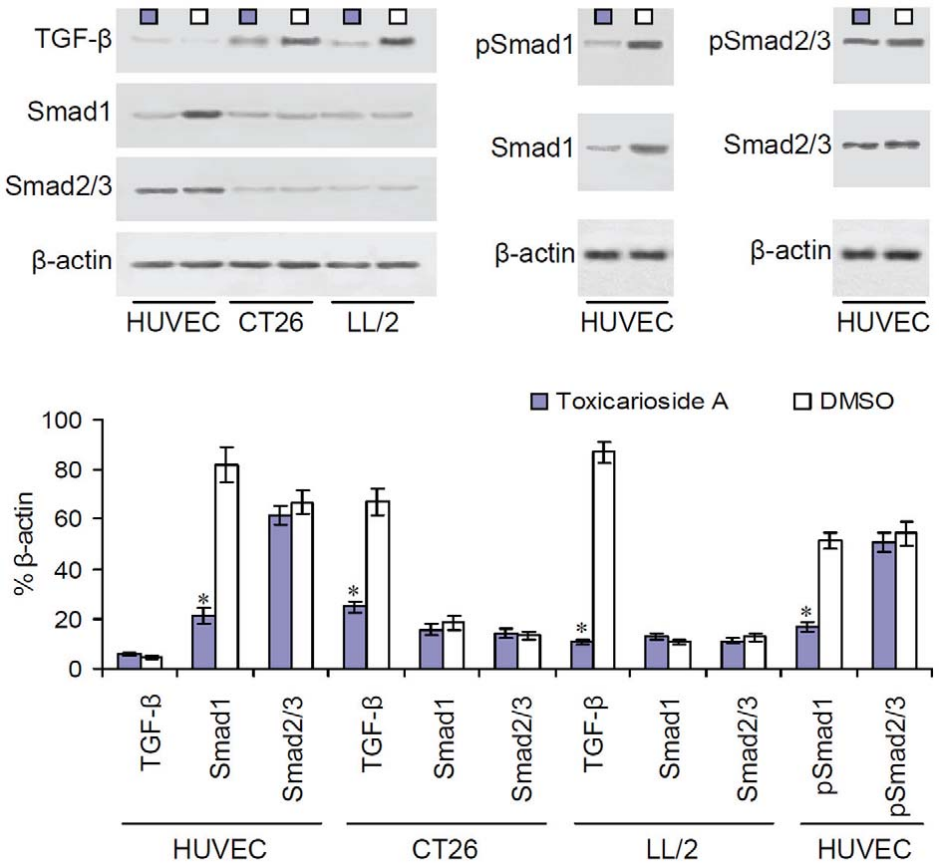
Inhibition of TGF-β expression and Smad proteins by toxicarioside A treatment. TGF-β and Smad proteins were detected by Western blot in HUVECs, CT26 cells, and LL/2 cells treated with toxicarioside A or DMSO. TGF-β expression was found to be attenuated in CT26 and LL/2 tumor cells treated with toxicarioside A but not in HUVECs relative to corresponding cells treated with DMSO. Smad1 and phosphorylated Smad1 (pSmad1) showed attenuation in HUVECs but not in CT26 and LL/2 tumor cells. Smad2/3 and pSmad2/3 remained unchanged in HUVECs, CT26 cells, and LL/2 tumor cells. Data are expressed as means ± SEM, *P<0.001 or less, relative to the DMSO-treated group.

Endoglin interacts with TGF-β receptor I isotypes ALK1 and ALK5, leading to phosphorylation of Smad1/5/8 and Smad2/3, respectively. For this reason, we next detected TGF-β, Smad1, Smad2/3, phosphorylated Smad1 (pSmad1), and phosphorylated Smad2/3 (pSmad2/3) by Western blot analysis. HUVECs, CT26 cells, and LL/2 cells were treated with toxicarioside A or DMSO for 24 hours and cell lysates were used to detect corresponding protein expression. TGF-β expression was not found in HUVECs, but it was found in CT26 and LL/2 tumor cells. Its expression could be significantly suppressed by toxicarioside A treatment (Figure 7, *P*<0.001). However, the suppression of TGF-β expression in CT26 and LL/2 induced by toxicarioside A did not cause changes in the expression of Smad1 and Smad2/3 (Figure 7). In addition, toxicarioside A treatment inhibited both Smad1 and pSmad1 expression, but not Smad2/3 and pSmad2/3 expression in HUVECs (Figure 7). These results indicate that toxicarioside A could affect endoglin expression in HUVECs and TGF-β expression in CT24 and LL/2 tumor cells. Toxicarioside A altered Smad1 signaling but not Smad2/3 signaling only in HUVECs, not in CT24 or LL/2 tumor cells.

### Inhibition of TGF-β signaling by endoglin interference

**Figure 8:**
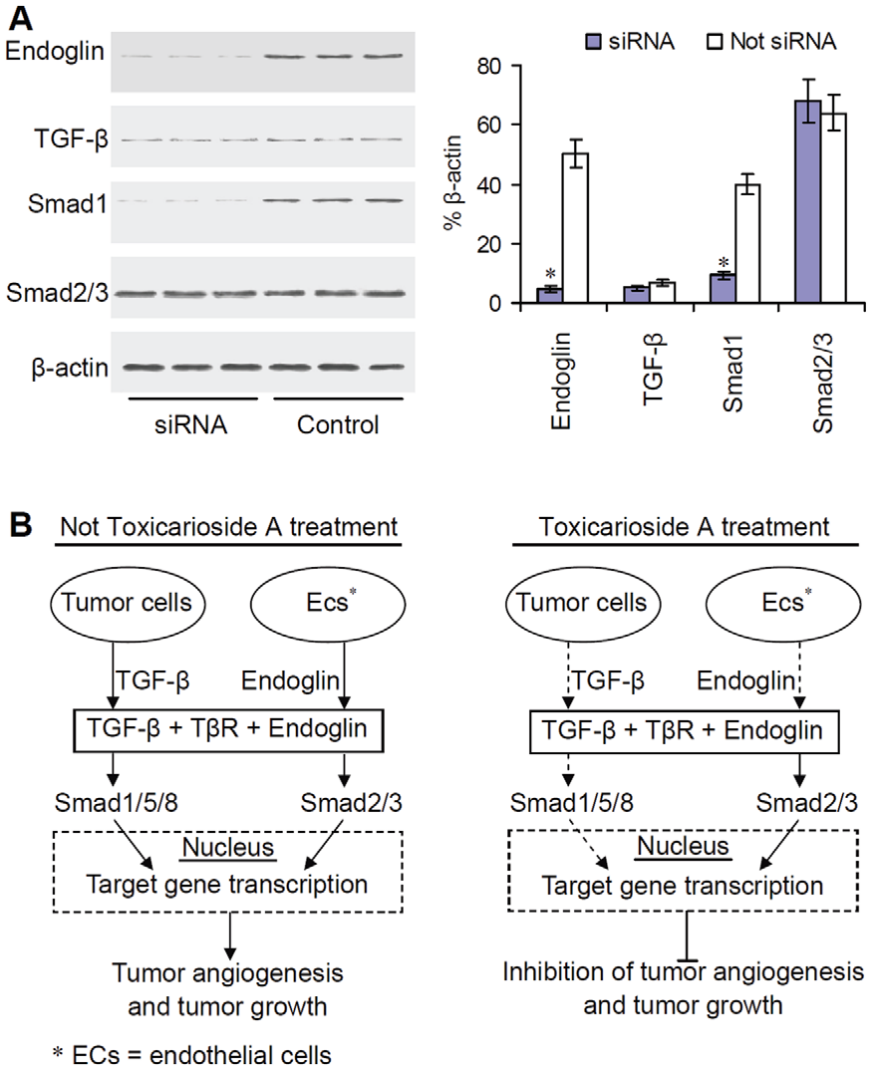
Inhibition of endoglin, TGF-β, and Smad protein expression by endoglin siRNA in HUVECs and model of antitumor mechanism of toxicarioside A. (**A**) Results of endoglin, TGF-β, and Smad protein expression detected by Western blot analysis. siRNA against endoglin induced significant attenuation of endoglin, TGF-β, and Smad1 in HUVECs treated with toxicarioside A, but Smad2/3 remained unchanged relative to DMSO treatment. Data are expressed as means ± SEM, *P<0.001 or less, relative to the control group. (**B**) Without toxicarioside A treatment, normal endoglin expression in endothelial cells and TGF-β expression in tumor cells caused a normal signaling cascade of ALK1-induced Smad1 and ALK5-induced Smad2/3 activation and tumor angiogenesis, which facilitated tumor growth. With toxicarioside A treatment, endoglin expression in endothelial cells and TGF-β expression in tumor cells were inhibited, causing attenuation of ALK1-induced Smad1 activation, which lead to inhibition of tumor angiogenesis and tumor growth.

Due to the role of endoglin in TGF-β signaling and decreased expression of endoglin in toxicarioside-A-treated HUVECs and TGF-β in the toxicarioside-A-treated CT26 and LL/2 tumor cells, we explored the roles played by endoglin and TGF-β in Smad signaling in HUVECs. We used siRNA against endoglin to attenuate endoglin protein levels in HUVECs. Endoglin protein levels were decreased by a factor of about 13 after siRNA interference against endoglin. The endoglin levels were 4.8±0.9 (% relative to β-actin) in the HUVECs treated with siRNA versus 62.6±6.2 in the control HUVECs (Figure 8A, *P*<0.001). Similar to the effects observed in the toxicarioside A-treated HUVECs, both Smad1 and Smad2/3 signaling were activated by stimulation of TGF-β, but only Smad1 activation was significantly decreased in the HUVECs treated with siRNA. It was not decreased in the control HUVECs, 9.3±1.2 (% relative to β-actin) and 2.7±0.7 versus 66.9±7.3 and 47.3±4.5, respectively (Figure 8A, *P*<0.001). In contrast, the protein levels of TGF-β were low and unaffected by endoglin siRNA interference. The protein level of TGF-β in the HUVECs treated with siRNA was 5.2±0.8 (% relative to β-actin) versus 6.8±1.1 in the control HUVECs (Figure 8A, *P*>0.05). These results indicated that the inhibition of the Smad1 singling in HUVECs by toxicarioside A treatment was the direct result of decreased endoglin expression and not related to other cellular changes, such as self-secreted TGF-β. However, exogenous TGF-β does appear to be needed for activation of Smad1 signaling.

## Discussion

*Antiaris toxicaria* is widespread throughout the tropical rainforests of southeastern Asia, and the plant is featured in many legends dating back to ancient times. It is especially famous for its poison, which has been used for arrows, darts, and blowdarts. Modern studies have isolated many chemically effective ingredients from its latex sap [35]. The most focus has been on the active complex mixture of cardenolide glycosides [17], [35]. In addition to the traditional effect of these cardenolides on the inhibition of the ubiquitous cell surface Na^+^ and K^+^-ATPase, over the past decade, there has been a substantial increase in the number of studies investigating the effects of cardiac glycosides on the growth of human malignant tumor cells and the possible molecular mechanisms behind them [9]–[12], [36]. In our previous works, we isolated three new cytotoxic cardenolides from the latex of *Antiaris toxicaria* and showed them to possess significant cytotoxicity against several human tumor cell lines *in vitro* [16], [17]. In the present study, we further investigated the *in vivo* anticancer activity and the potential molecular mechanism behind toxicarioside A, one of the cytotoxic cardenolides that we isolated from the latex of *Antiaris toxicaria* in our laboratory. We established *in vivo* tumor models of CT26 colorectal carcinoma and LL/2 Lewis lung carcinoma in BALB/c and C57BL/6 mice, respectively. Relative to the DMSO control mice, we found that tumor growth and angiogenesis were significantly suppressed in mice treated with the optimum effective dose of toxicarioside A. *In situ* detection of endoglin expression on the tumor vessels by immunofluorescence showed that toxicarioside A treatment lead to significant inhibition of endoglin expression on the tumor vessels relative to the control DMSO treatment. In addition, endoglin expression was decreased in tumor tissues treated with toxicarioside A and *in vitro* cultured HUVEC supplemented with toxicarioside A in the culture media, but not in CT26 and LL/2 tumor cells. These results indicate that endoglin is mainly expressed in the endothelial cells and toxicarioside A can suppress the endoglin expression in the endothelial cells.

Endoglin works as an auxiliary regulatory component by formation of a heteromeric complex with the TGF-β receptor, by which it modulates the signaling of distinct TGF-β receptor I isotypes ALK-1 and ALK-5 [21], [22]. Considering that TGF-β is one of the endoglin's ligands, we investigated whether HUVECs or tumor cells could express TGF-β and whether toxicarioside A would affect TGF-β expression. In the present study, we used Western blot analysis to detect TGF-β expression in HUVECs and tumor cells. Although small amounts of TGF-β were detected in HUVECs, there was no significant difference between HUVECs treated with toxicarioside A and control DMSO. In contrast, 60–80% (relative to β-actin) expression of TGF-β was observed in the CT26 and LL/2 tumor cells, and its expression was found to be significantly suppressed by toxicarioside A treatment. Tumor cells are major cell types within the tumor mass, and endothelial cells mainly exist in the newly formed blood vessels. Therefore, it is easy to conceive that the TGF-β secreted by the tumor cells will act with the endoglin in the endothelial cells to promote tumor angiogenesis, which will further facilitate tumor growth and metastasis.

A series of preclinical and clinical studies have indicated that endoglin is a hallmark molecule of tumor angiogenesis. It is over-expressed and up-regulated in tumor-associated angiogenic vasculature relative to normal tissue vasculature [37]–[41]. Although the exact mechanism by which endoglin induces tumor angiogenesis remains to be illustrated, it is well accepted that endoglin acts as a positive regulator of ALK1 signaling and a negative regulator of ALK5 signaling in endothelial cells [42]–[43]. Endoglin initiates its intracellular signaling cascade through phosphorylation of specific Smad proteins by which further signals are transduced into the nucleus and series genes are regulated to be transcribed after formation of the activated heteromeric complex with TGF-β receptor I isotypes ALK-1 and ALK-5 [21], [22], [42], [43]. In the present study, *in vivo* results showed that toxicarioside A treatment could significantly suppress tumor growth and inhibit tumor angiogenesis in CT26 and LL/2 tumor models. The expression of endoglin in the endothelial cells and expression of TGF-β in the tumor cells were significantly inhibited by toxicarioside A treatment. The detection of Smad proteins showed that Smad1 and its activated counterpart, pSmad1, were significantly attenuated in the HUVECs, whereas Smad2/3 and pSmad2/3 were not affected. Therefore, it can be concluded that attenuation of the ALK1-induced Smad1/5/8 signaling pathway through suppression of endoglin and TGF-β expression in the endothelial cells and in the tumor cells is responsible for the effects on the *in vivo* inhibition of tumor growth and tumor angiogenesis by toxicarioside A treatment (Figure 8B).

To determine if the antitumor effects of toxicarioside A were the direct result of a primary effect on endoglin attenuation in the endothelial cells but not directly related to the TGF-β in the tumor cells, endoglin expression was selectively attenuated in HUVECs using siRNA. Similar the results observed in the HUVECs treated with toxicarioside A, endoglin protein levels were decreased and Smad signaling was altered in the HUVECs transfected with endoglin siRNA. Smad1 but not Smad2/3 protein levels were significantly decreased, suggesting that endoglin plays a role in the attenuation of endothelial tumor growth and tumor angiogenesis.

In the present study, we found that toxicarioside A inhibited endoglin expression mainly in HUVECs and inhibited TGF-β expression in tumor cells, which lead to suppress tumor growth through inhibition of tumor angiogenesis. In addition, we still found that toxicarioside A could significantly suppress cell proliferation, migration and invasion in HUVECs, CT26 and LL/2 cells. Moreover, toxicarioside A treatment did not induce cell apoptosis and cause abnormal expression of other membrane proteins, such as CD31 and MHC I on HUVECs, CT26 and LL/2 cells. These data suggest that toxicarioside A specifically acts on the suppression of endoglin expression. However, our present data can not identify the underlying mechanisms by which toxicarioside A treatment causes such cell type-specific expression of endoglin. We suppose that there may be a common mechanism by which toxicarioside A regulate both endoglin and TGF-β transcription expression. Therefore, further studies are needed in order to unveil the possible mechanism related to endoglin and TGF-β expression inhibited by toxicarioside A.

In summary, our results indicate that toxicarioside A can suppress tumor growth and tumor angiogenesis by attenuating the endoglin expression in endothelial cells and TGF-β in tumor cells. The attenuated expression of endoglin and TGF-β together further induce inhibition of cascade activation of ALK1 (Smad1/5/8) in endothelial cells, resulting in decreased tumor angiogenesis and suppression of tumor growth.

## References

[1] Ottensmeyer FP, Schmidt EE, Jack T, Powell J (1972) Molecular architecture: the optical treatment of dark field electron micrographs of atoms. J Ultrastruct Res 40: 546–555. 5055812 10.1016/s0022-5320(72)80042-x

[2] Shrestha T, Kopp B, Bisset NG (1992) The Moraceae-based dart poisons of South America. Cardiac glycosides of Maquira and Naucleopsis species. J Ethnopharmacol 37: 129–143. 1434687 10.1016/0378-8741(92)90071-x

[3] Fujimoto Y, Suzuki Y, Kanaiwa T, Amiya T, Hoshi K, et al (1983) Studies on the Indonesian Antiaris toxicaria sap. J Pharmacobiodyn 6: 128–135. 6306201 10.1248/bpb1978.6.128

[4] Kopp B, Bauer WP, Bernkop-Schnurch A (1992) Analysis of some Malaysian dart poisons. J Ethnopharmacol 36: 57–62. 1501494 10.1016/0378-8741(92)90061-u

[5] Shi LS, Liao YR, Su MJ, Lee AS, Kuo PC, et al (2010) Cardiac glycosides from Antiaris toxicaria with potent cardiotonic activity. J Nat Prod 73: 1214–1222. 20553004 10.1021/np9005212 PMC2917517

[6] Gheorghiade M, van Veldhuisen DJ, Colucci WS (2006) Contemporary use of digoxin in the management of cardiovascular disorders. Circulation 113: 2556–2564. 16735690 10.1161/CIRCULATIONAHA.105.560110

[7] Hamad E, Mather PJ, Srinivasan S, Rubin S, Whellan DJ, et al (2007) Pharmacologic therapy of chronic heart failure. Am J Cardiovasc Drugs 7: 235–248. 17696565 10.2165/00129784-200707040-00002

[8] Wang Z, Zheng M, Li Z, Li R, Jia L, et al (2009) Cardiac glycosides inhibit p53 synthesis by a mechanism relieved by Src or MAPK inhibition. Cancer Res 69: 6556–6564. 19679550 10.1158/0008-5472.CAN-09-0891 PMC2728080

[9] Frese S, Frese-Schaper M, Andres AC, Miescher D, Zumkehr B, et al (2006) Cardiac glycosides initiate Apo2L/TRAIL-induced apoptosis in non-small cell lung cancer cells by up-regulation of death receptors 4 and 5. Cancer Res 66: 5867–5874. 16740726 10.1158/0008-5472.CAN-05-3544

[10] McConkey DJ, Lin Y, Nutt LK, Ozel HZ, Newman RA (2000) Cardiac glycosides stimulate Ca2+ increases and apoptosis in androgen-independent, metastatic human prostate adenocarcinoma cells. Cancer Res 60: 3807–3812. 10919654

[11] Lin J, Denmeade S, Carducci MA (2009) HIF-1alpha and calcium signaling as targets for treatment of prostate cancer by cardiac glycosides. Curr Cancer Drug Targets 9: 881–887. 20025575 10.2174/156800909789760249

[12] Newman RA, Yang P, Pawlus AD, Block KI (2008) Cardiac glycosides as novel cancer therapeutic agents. Mol Interv 8: 36–49. 18332483 10.1124/mi.8.1.8

[13] Prassas I, Karagiannis GS, Batruch I, Dimitromanolakis A, Datti A, et al (2011) Digitoxin-induced cytotoxicity in cancer cells is mediated through distinct kinase and interferon signaling networks. Mol Cancer Ther 10: 2083–2093. 21859838 10.1158/1535-7163.MCT-11-0421

[14] Mijatovic T, Van Quaquebeke E, Delest B, Debeir O, Darro F, et al (2007) Cardiotonic steroids on the road to anti-cancer therapy. Biochim Biophys Acta 1776: 32–57. 17706876 10.1016/j.bbcan.2007.06.002

[15] Prassas I, Diamandis EP (2008) Novel therapeutic applications of cardiac glycosides. Nat Rev Drug Discov 7: 926–935. 18948999 10.1038/nrd2682

[16] Dong WH, Mei WL, Zhao YX, Zeng YB, Wang H, et al (2011) A new drimane sesquiterpenoid glycoside from the seeds of Antiaris toxicaria. J Asian Nat Prod Res 13: 561–565. 21623521 10.1080/10286020.2011.573479

[17] Dai HF, Gan YJ, Que DM, Wu J, Wen ZC, et al (2009) Two new cytotoxic cardenolides from the latex of Antiaris toxicaria. J Asian Nat Prod Res 11: 832–837. 20183332 10.1080/10286020903164285

[18] Li YN, Huang FY, Mei WL, Dai HF, Guo JL, et al (2012) Toxicarioside A, isolated from tropical Antiaris toxicaria, blocks endoglin/TGF-beta signaling in a bone marrow stromal cell line. Asian Pac J Trop Med 5: 91–97. 22221748 10.1016/S1995-7645(12)60002-9

[19] Guo JL, Zheng SJ, Li YN, Jie W, Hao XB, et al (2012) Toxicarioside A inhibits SGC-7901 proliferation, migration and invasion via NF-κB/bFGF signaling. World J Gastroenterol 18: 1602–1609. 22529688 10.3748/wjg.v18.i14.1602 PMC3325525

[20] Barbara NP, Wrana JL, Letarte M (1999) Endoglin is an accessory protein that interacts with the signaling receptor complex of multiple members of the transforming growth factor-beta superfamily. J Biol Chem 274: 584–594. 9872992 10.1074/jbc.274.2.584

[21] Blanco FJ, Santibanez JF, Guerrero-Esteo M, Langa C, Vary CP, et al (2005) Interaction and functional interplay between endoglin and ALK-1, two components of the endothelial transforming growth factor-beta receptor complex. J Cell Physiol 204: 574–584. 15702480 10.1002/jcp.20311

[22] Lebrin F, Deckers M, Bertolino P, Ten Dijke P (2005) TGF-beta receptor function in the endothelium. Cardiovasc Res 65: 599–608. 15664386 10.1016/j.cardiores.2004.10.036

[23] Itoh S, Itoh F, Goumans MJ, Ten Dijke P (2000) Signaling of transforming growth factor-beta family members through Smad proteins. Eur J Biochem 267: 6954–6967. 11106403 10.1046/j.1432-1327.2000.01828.x

[24] Fonsatti E, Nicolay HJ, Altomonte M, Covre A, Maio M (2010) Targeting cancer vasculature via endoglin/CD105: a novel antibody-based diagnostic and therapeutic strategy in solid tumours. Cardiovasc Res 86: 12–19. 19812043 10.1093/cvr/cvp332

[25] Dallas NA, Samuel S, Xia L, Fan F, Gray MJ, et al (2008) Endoglin (CD105): a marker of tumor vasculature and potential target for therapy. Clin Cancer Res 14: 1931–1937. 18381930 10.1158/1078-0432.CCR-07-4478

[26] Jiao JG, Li YN, Wang H, Liu Q, Cao JX, et al (2006) A plasmid DNA vaccine encoding the extracellular domain of porcine endoglin induces anti-tumour immune response against self-endoglin-related angiogenesis in two liver cancer models. Dig Liver Dis 38: 578–587. 16777500 10.1016/j.dld.2006.04.014

[27] Nassiri F, Cusimano MD, Scheithauer BW, Rotondo F, Fazio A, et al (2011) Endoglin (CD105): a review of its role in angiogenesis and tumor diagnosis, progression and therapy. Anticancer Res 31: 2283–2290. 21737653

[28] Takahashi N, Haba A, Matsuno F, Seon BK (2001) Antiangiogenic therapy of established tumors in human skin/severe combined immunodeficiency mouse chimeras by anti-endoglin (CD105) monoclonal antibodies, and synergy between anti-endoglin antibody and cyclophosphamide. Cancer Res 61: 7846–7854. 11691802

[29] Tan GH, Wei YQ, Tian L, Zhao X, Yang L, et al (2004) Active immunotherapy of tumors with a recombinant xenogeneic endoglin as a model antigen. Eur J Immunol 34: 2012–2021. 15214049 10.1002/eji.200424933

[30] Huang FY, Mei WL, Li YN, Tan GH, Dai HF, et al (2012) The antitumour activities induced by pegylated liposomal cytochalasin D in murine models. Eur J Cancer 48: 2260–2269. 22257793 10.1016/j.ejca.2011.12.018

[31] Zhong NT, Huang FY, Tan GH, Jiao JG, Lin YZ, et al.. (2010) Effect of hepatocyte growth factor signaling pathway activation on Plasmodium berhei infection. Asian Pac J Trop Med 2010.

[32] Apte RS, Niederkorn JY, Mayhew E, Alizadeh H (2001) Angiostatin produced by certain primary uveal melanoma cell lines impedes the development of liver metastases. Arch Ophthalmol 119: 1805–1809. 11735791 10.1001/archopht.119.12.1805

[33] Santibanez JF, Letamendia A, Perez-Barriocanal F, Silvestri C, Saura M, et al (2007) Endoglin increases eNOS expression by modulating Smad2 protein levels and Smad2-dependent TGF-beta signaling. J Cell Physiol 210: 456–468. 17058229 10.1002/jcp.20878

[34] O'Connor JC, Farach-Carson MC, Schneider CJ, Carson DD (2007) Coculture with prostate cancer cells alters endoglin expression and attenuates transforming growth factor-beta signaling in reactive bone marrow stromal cells. Mol Cancer Res 5: 585–603. 17579118 10.1158/1541-7786.MCR-06-0408

[35] Jiang MM, Dai Y, Gao H, Zhang X, Wang GH, et al (2008) Cardenolides from Antiaris toxicaria as potent selective Nur77 modulators. Chem Pharm Bull (Tokyo) 56: 1005–1008. 18591820 10.1248/cpb.56.1005

[36] Sreenivasan Y, Sarkar A, Manna SK (2003) Oleandrin suppresses activation of nuclear transcription factor-kappa B and activator protein-1 and potentiates apoptosis induced by ceramide. Biochem Pharmacol 66: 2223–2239. 14609747 10.1016/j.bcp.2003.07.010

[37] Bodey B, Bodey B Jr, Siegel SE, Kaiser HE (1998) Over-expression of endoglin (CD105): a marker of breast carcinoma-induced neo-vascularization. Anticancer Res 18: 3621–3628. 9858949

[38] Bodey B, Bodey B Jr, Siegel SE, Kaiser HE (1998) Immunocytochemical detection of endoglin is indicative of angiogenesis in malignant melanoma. Anticancer Res 18: 2701–2710. 9703932

[39] Seon BK, Matsuno F, Haruta Y, Kondo M, Barcos M (1997) Long-lasting complete inhibition of human solid tumors in SCID mice by targeting endothelial cells of tumor vasculature with antihuman endoglin immunotoxin. Clin Cancer Res 3: 1031–1044. 9815781

[40] Burrows FJ, Derbyshire EJ, Tazzari PL, Amlot P, Gazdar AF, et al (1995) Up-regulation of endoglin on vascular endothelial cells in human solid tumors: implications for diagnosis and therapy. Clin Cancer Res 1: 1623–1634. 9815965

[41] Wang JM, Kumar S, Pye D, van Agthoven AJ, Krupinski J, et al (1993) A monoclonal antibody detects heterogeneity in vascular endothelium of tumours and normal tissues. Int J Cancer 54: 363–370. 8509210 10.1002/ijc.2910540303

[42] Zhang L, Magli A, Catanese J, Xu Z, Kyba M, et al (2011) Modulation of TGF-beta signaling by endoglin in murine hemangioblast development and primitive hematopoiesis. Blood 118: 88–97. 21602526 10.1182/blood-2010-12-325019 PMC3139390

[43] Scherner O, Meurer SK, Tihaa L, Gressner AM, Weiskirchen R (2007) Endoglin differentially modulates antagonistic transforming growth factor-beta1 and BMP-7 signaling. J Biol Chem 282: 13934–13943. 17376778 10.1074/jbc.M611062200

